# Sleep-related respiratory disruptions and laterodorsal tegmental nucleus in a mouse model of Parkinson’s disease

**DOI:** 10.1016/j.isci.2024.111251

**Published:** 2024-10-24

**Authors:** Nicole C. Miranda, Luiz M. Oliveira, Thiago S. Moreira, Jan-Marino Ramirez, Franck Kalume, Ana C. Takakura

**Affiliations:** 1 Department of Pharmacology, Instituto de Ciencias Biomedicas, Universidade de Sao Paulo, SP, São Paulo 05508-000, SP, Brazil; 2 Center for Integrative Brain Research, Seattle Children’s Research Institute, 1900 9th Avenue, Seattle, WA 98101, USA; 3 Department of Physiology and Biophysics, Instituto de Ciencias Biomedicas, Universidade de Sao Paulo, SP, São Paulo 05508-000, SP, Brazil; 4 Department of Neurological Surgery, University of Washington, 1900 9th Avenue, Seattle, WA 98101, USA; 5 Department of Pediatrics, University of Washington, 1900 9th Avenue, Seattle, WA 98101, USA

**Keywords:** Disease, Biological sciences, Neuroscience

## Abstract

Parkinson’s disease (PD) is a chronic neurodegenerative disorder affecting the motor system, with non-classic symptoms such as sleep disturbances and respiratory dysfunctions. These issues reflect a complex pathophysiological interaction that severely impacts quality of life. Using a 6-hydroxydopamine (6-OHDA) mouse model of PD, we investigated these connections by analyzing sleep patterns and respiratory parameters during non-rapid eye movement (NREM) and rapid eye movement (REM) sleep. Our findings revealed altered breathing, including reduced respiratory frequency and increased apneas during both NREM and REM. To address these abnormalities, we employed chemogenetic stimulation of cholinergic neurons in the laterodorsal tegmental nucleus (LDTg), a key region for sleep-wake regulation and respiratory modulation. This intervention normalized respiratory function. These results highlight the critical role of LDTg cholinergic neurons in the coordinating sleep and breathing, suggesting that targeting these neurons could offer a therapeutic strategy for managing PD-related respiratory complications.

## Highlights

• Sleep and respiratory changes are linked with neurodegeneration in the 6-OHDA model
• 6-OHDA reduces respiratory frequency, apneas in sleep, and LDTg cholinergic neurons
• LDTg cholinergic neurons regulate respiratory frequency/apneas in sleep-wake cycles
• Modulating LDTg neurons may help manage respiratory complications in PD

## Graphical abstract

**Figure:**
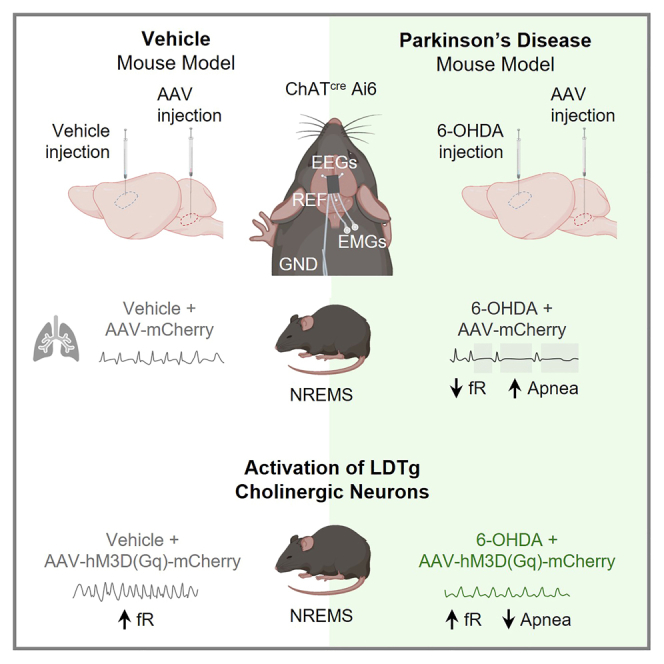

## Introduction

Parkinson’s disease (PD) is characterized by the progressive degeneration of dopaminergic neurons within the substantia nigra pars compacta (SNpc). As the disease advances, individuals may manifest motor symptoms, including resting tremor, muscle rigidity, bradykinesia, and postural changes.^1^^,^^2^^,^^3^ However, the paradigm of PD extends beyond these hallmark manifestations. The progression of PD is associated with a spectrum of non-classic symptoms, encompassing challenges such as impaired memory, depressive states, sleep disturbances, and autonomic and respiratory disorders.^4^^,^^5^ Some of these symptoms may precede the obvious manifestation of motor symptoms and may contribute to the overall progression of the disease, underscoring the complexity of PD. Unraveling the diverse ways in which both motor and non-motor aspects impact an individual’s health is of paramount importance for understanding PD.

Sleep disturbances are among the various symptoms observed even before the emergence of classical disease symptoms. The most prevalent among these disturbances is rapid eye movement (REM) sleep behavior disorder (RBD), a condition characterized by individuals acting out vivid dreams during REM sleep. This often involves vocalizations and sudden, potentially violent arm and leg movements, which are caused by a loss of the normal atonia that typically occurs during this phase of the sleep cycle.^6^^,^^7^^,^^8^^,^^9^ Beyond RBD, a spectrum of other symptoms such as daytime sleepiness, insomnia, nocturia, restless legs syndrome, and sleep-related breathing disturbances affects approximately 90% of individuals with PD. These disruptions in sleep patterns not only contribute to the complexity of PD but may also serve as predictive indicators for a more pronounced impact on non-motor symptoms.^10^^,^^11^^,^^12^^,^^13^

As the condition progresses, individuals with PD may also develop respiratory changes, including those related to sleep, such as obstructive sleep apnea.^14^^,^^15^^,^^16^^,^^17^^,^^18^^,^^19^ Recent studies suggest that these respiratory and sleep-related alterations result from the degeneration of brain regions responsible for the control of these functions. Specifically, the degeneration of the retrotrapezoid nucleus (RTN), nucleus of the solitary tract (NTS), and pre-Bötzinger complex (preBötC) has been implicated in alterations of respiratory control and chemoreception associated with PD.^20^^,^^21^^,^^22^^,^^23^^,^^24^

The laterodorsal tegmental area (LDTg) plays a critical role in regulating states of sleep and wakefulness, including motor atonia during REM sleep.^25^^,^^26^^,^^27^^,^^28^ The LDTg is comprised mainly of cholinergic cells and projects to various crucial brain areas, such as the ventral tegmental area (VTA), substantia nigra (SNc), amygdala, dorsal raphe, nucleus accumbens, striatum, and thalamus.^27^ However, its specific role in coordinating sleep and breathing, especially in comparison to the nearby pedunculopontine nucleus (PPTg), remains poorly understood. The anatomical proximity of the LDTg and the PPTg has presented challenges in discerning their distinct functions using conventional lesioning and pharmacological approaches. Additionally, it is known that cholinergic neurons in individuals with PD degenerate and this cell loss has been implicated in the mechanisms of motor impairments.^29^^,^^30^^,^^31^

In the present study, we employed an experimental model of PD, induced by the injection of 6-hydroxydopamine (6-OHDA: a structural analogue of catecholamines, dopamine, and noradrenaline, that exerts its toxic effects on catecholaminergic neurons) into the striatum, to explore the possibility that the LDTg is also a critical contributor to breathing dysfunction in PD. Using transgenic mice and viral vectors approaches to specifically characterize and manipulate the LDTg, we demonstrated its significant impact on sleep and breathing coordination in healthy mice. This represents a contribution to the field. Furthermore, we showed that the degeneration of cholinergic neurons in the LDTg significantly contributes to sleep and respiratory dysfunction in PD.

## Results

### Sleep-wake alterations in 6-OHDA PD-animals

**Figure 1:**
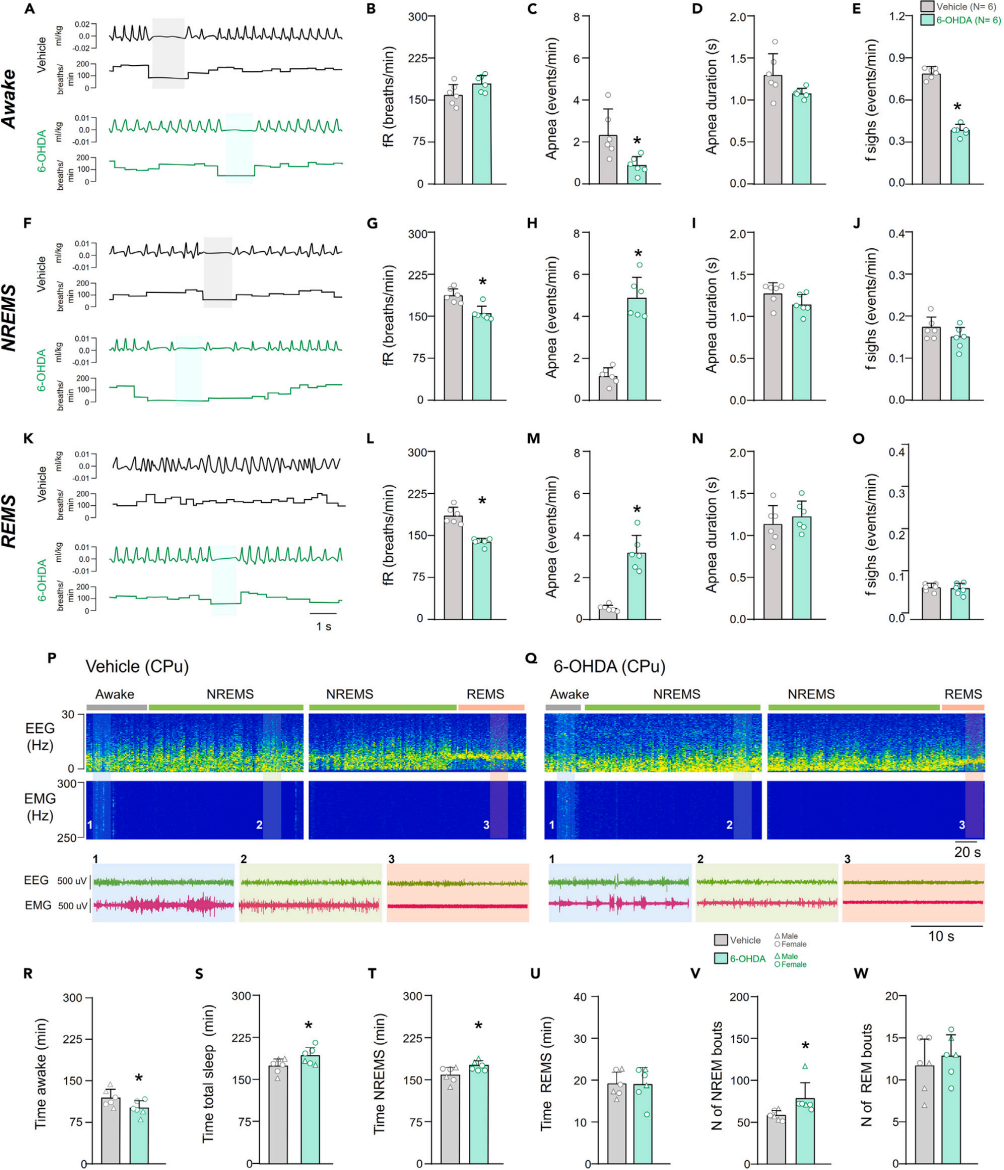
Respiratory changes in different phases of the sleep-wake cycle and characterization of the sleep-wake cycle in mice in mice receiving bilateral injections of either vehicle or 6-OHDA into the CPu Data are represented as mean ± SD. (A–E) In wake, examples of the recording of basal ventilation (A), graphs showing the quantification of respiratory frequency (B), the quantification of the number of apneas (C), the quantification of apnea duration (D) and the quantification of sighs frequency (E) after 10 days of vehicle or 6-OHDA injections in the CPu. (F–J) In NREM sleep, examples of the recording of basal ventilation (F), graphs showing the quantification of respiratory frequency (G), the quantification of the number of apneas (H), the quantification of apnea duration (I) and the quantification of sighs frequency (J) after 10 days of vehicle or 6-OHDA injections in the CPu. (K–O) In REM sleep, examples of the recording of basal ventilation (K), graphs showing the quantification of respiratory frequency (L), the quantification of the number of apneas (M), the quantification of apnea duration (N) and the quantification of sighs frequency (O) after 10 days of vehicle or 6-OHDA injections in the CPu. (P and Q) Recordings displaying the difference in electroencephalographic and electromyographic waves in mice receiving vehicle (P) or 6-OHDA into the CPu (Q). (R–W) Comparative data related to total wake time (R), total sleep time (S), NREM sleep time (T), REM sleep time (U), and the number of NREM (V) and REM (W) sleep episodes in animals receiving vehicle or 6-OHDA into the CPu, the triangle symbols represent male mice, and the circles represent female mice. Abbreviations: NREMS, NREM sleep; REMS, REM sleep; EEG, electroencephalogram; EMG, electromyogram. Statistical analysis: ∗different from vehicle, *p* < 0.05, t test.

Several types of sleep disturbances are observed in humans affected by PD.^11^^,^^19^^,^^32^^,^^33^ However, there are still limited studies in this experimental mouse model of PD induced by bilateral injections of 6-OHDA. Therefore, we investigated the sleep patterns by electrophysiologically derived observations (see STAR Methods for details) of these animals during the light phase over 5 h (Figures 1P and 1Q). During this time period we found decreased wakefulness time in PD-induced animals (PD: 102.5 ± 13.4 vs. Vehicle (Control group): 121.2 ± 15.3 min, *p* = 0.0481, t-test, t = 2.251, df = 10) (Figure 1R). Additionally, PD-induced animals exhibited longer total sleep time (PD: 194.5 ± 15.3 vs. Control: 176.0 ± 12.9 min, *p* = 0.0483, t-test, t = 2.248, df = 10) (Figure 1S) and non-rapid eye movement (NREM) sleep time (PD: 175.9 ± 8.6 vs. Control 159.7 ± 13.9 min, *p* = 0.0359, t-test, t = 2.423, df = 10) (Figure 1T) compared to controls, with no alterations in total REM sleep time (PD: 19.1 ± 4.1 vs. Control: 19.1 ± 2.8 min, *p* = 0.9894, t-test, t = 0.01363, df = 10) (Figure 1U). Additionally, the counts of NREMS episodes revealed that PD-induced animals had a higher number of NREM bouts (PD: 78.0 ± 19.2 vs. Control: 58.1 ± 5.9 number of NREMS episodes, *p* = 0.0368, t-test, t = 2.408, df = 10) (Figure 1V) and without change in the number of REMS bouts (PD: 12.8 ± 2.5 vs. Control: 11.6 ± 3.2 number of REMS episodes, *p* = 0.5020, t-test, t = 0.6965, df = 10) (Figure 1W). These findings indicate that PD-induced animals generated by injection of 6-OHDA into striatum exhibit more episodes and time in NREMS, without changes in REMS (Figures 1P–1W).

We also decided to differentiate and compared sleep parameters between male and female mice and did not observe any significant differences in any of the variables analyzed (Figures 1R–1W).

### Respiratory alterations in the different phases of the sleep-wake cycle in PD-animals

One characteristic of the 6-OHDA PD model is a reduction in resting respiratory frequency, but it remains unclear during which phase of the sleep-wake cycle this alteration occurs. During wakefulness, the respiratory frequency did not show statistical significance (PD: 181.5 ± 14.7 vs. Control: 161.5 ± 19.3 breaths/min, *p* = 0.0702, t = 2.027, df = 10) (Figures 1A and 1B), while a reduction in respiratory frequency occurred in both NREM sleep (PD: 158.0 ± 13.1 vs. Control: 190.8 ± 12.9 breaths/min, *p* = 0.0014, t = 4.356, df = 10) (Figures 1F and 1G) and REM sleep (PD: 140.7 ± 6.6 vs. Control: 188.5 ± 15.0 breaths/min, *p* < 0.0001, t = 7.124, df = 10) (Figures 1K and 1L). Furthermore, we observed an increase in the number of apneas during NREMS (PD: 4.9 ± 0.9 vs. Control: 1.2 ± 0.4 apneas/min, *p* < 0.0001, t = 8.622, df = 10) (Figure 1H) and REMS (PD: 3.2 ± 0.8 vs. Control: 0.5 ± 0.1 apneas/min, *p* < 0.0001, t = 7.667, df = 10) (Figure 1M), with a decrease during wakefulness (PD: 0.9 ± 0.4 vs. Control: 2.4 ± 1.2 apneas/min, *p* = 0.0144, t = 2.954, df = 10) (Figure 1C). There were no significant changes in the duration of apneas in any phase of the sleep-wake cycle (Figures 1D, 1I, and 1N). In relation to the quantity of sighs, we observed a decrease during wakefulness (PD: 0.3 ± 0.03 vs. Control: 0.7 ± 0.04 sigh/min, *p* < 0.0001, t = 17.99, df = 10) (Figure 1E) and no difference in NREMS or REMS (PD: 0.1 ± 0.02 vs. Control: 0.1 ± 0.02 sigh/min, *p* = 0.1449, t = 1.581, df = 10 in NREMS and PD: 0.07 ± 0.01 vs. Control: 0.07 ± 0.01 sigh/min, *p* = 0.5556, t = 0.609, df = 10 in REMS) (Figures 1J and 1O).

### Characterization of distinct subpopulations and cholinergic projections of LDTg neurons in WT mice

As a first step to studying the role of the LDTg in driving sleep-related respiratory dysfunctions in the PD-induced mouse model, we examined the diverse neuronal population within the LDTg. The LDTg region contains not only cholinergic neurons but also glutamatergic and GABAergic neurons. Given this cellular diversity, we set out to phenotypically characterize the different neuronal populations within this region. To quantify the neuronal subtypes in the LDTg area, we employed a genetic strategy. We crossed floxed-STOP ZsGreen reporter mice with homozygous Vglut2^cre^, Vgat^cre^, or ChAT^cre^ mice, enabling the permanent labeling of cells expressing Vglut2, Vgat, and ChAT with a robust fluorescent marker, primarily cell bodies.

**Figure 2:**
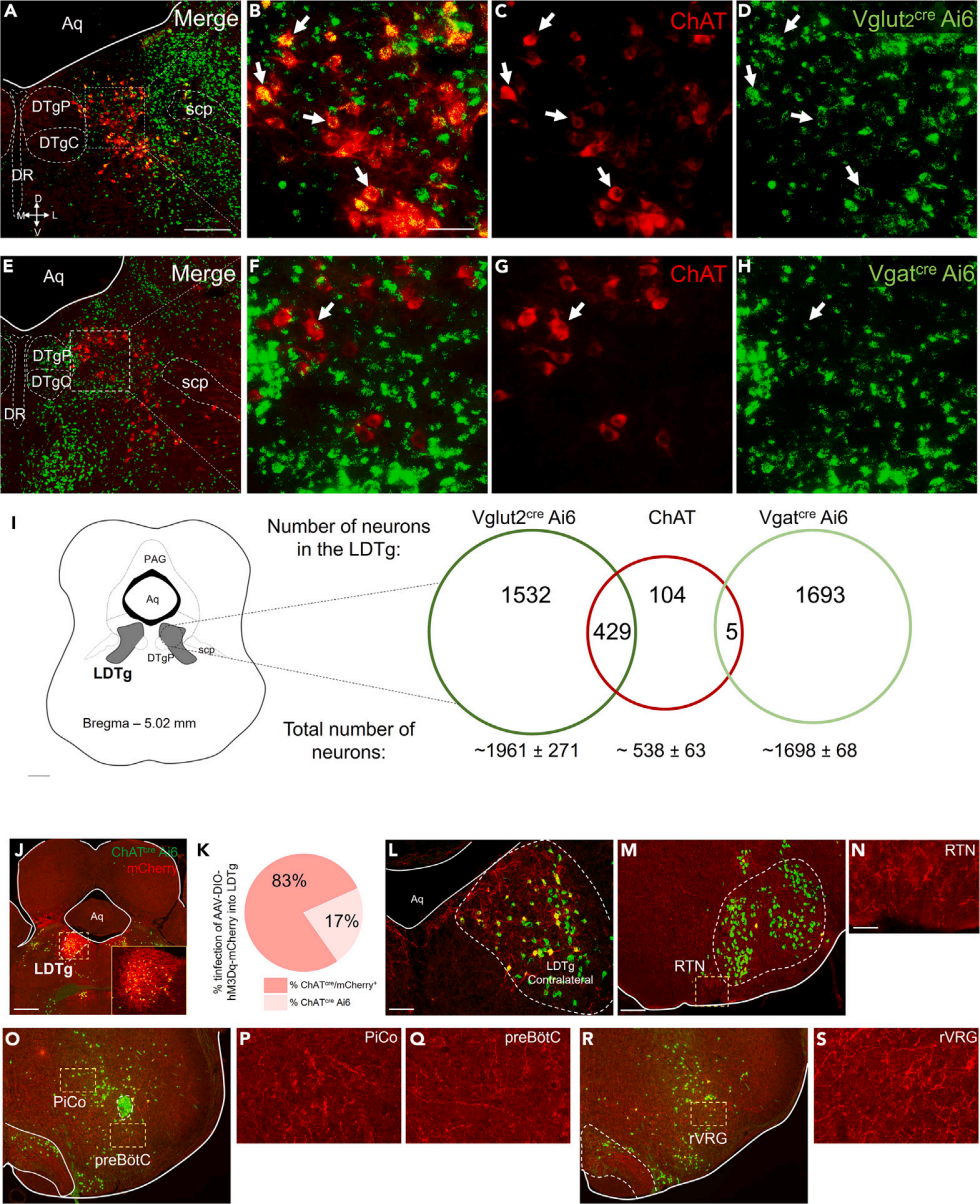
Characterization of LDTg neurons in Vglut2^cre^ Ai6 and Vgat^cre^ Ai6 double-labeled mice for ChAT Data are represented as mean ± SD (A–D) Photomicrographs showing immunofluorescence for Vglut2^cre^/ChAT (A and B), ChAT (C) Vglut2^cre^ Ai6 (D). (E–H) Photomicrographs showing immunofluorescence for Vgat^cre^/ChAT (E and F), ChAT (G) Vgat^cre^ Ai6 (H). (I) Schematic drawing demonstrating LDTg region and the number of neurons Vglut2^cre^ Ai6 in dark green, ChAT neurons in red and Vgat^cre^ Ai6 in light green. (J) Injection site to AAV-hM3Dq-mCherry into LDTg in mice ChAT^cre^ Ai6. (K) Percentage of infection of the AAV-hM3Dq-mCherry into LDTg (*N* = 4). (L–S) Photomicrographs showing the varicosities of the anterograde tracer in the LDTg contraleral region (L), RTN (M) and higher magnification (N), PiCo and preBӧtC (O) and higher magnification of PiCo (P) and preBӧtC (Q) and rVRG (R) and higher magnification (S). Abbreviations: scp, superior cerebellar peduncle; DTgC, dorsal tegmental nucleus, central part; DTgP, dorsal tegmental nucleus, pericentral part; DR, dorsal raphe nucleus; LDTg, laterodorsal tegmental nucleus; PAG, periaqueductal gray; Aq, aqueduct; SNpc, compact substantia nigra; VTA, ventral tegmental area; RTN, retrotrapezoid nucleus; PiCo, postinspiratory complex; preBӧtC, pre-Bötzinger complex; rVRG, rostral division of ventral respiratory group. Scale bars: A = 500 μm (applies to A, E) I, J, M, O, R) and B = 100 μm (applies to C and D, F and H), I and J = 500 μm, M = 500 μm (applies to O and R), N = 100 μm (applies to P, Q, and S).

Quantification of labeled cells revealed approximately 1,961 glutamatergic neurons, 538 cholinergic neurons, and 1,698 GABAergic neurons in LDTg. Notably, our analyses indicated that a substantial portion (∼22%) of glutamatergic neurons in the LDTg were also cholinergic. In contrast only a negligible portion of these neurons co-labeled as GABAergic (Vgat^+^) (Figures 2A–2I).

Furthermore, given the absence of literature describing whether cholinergic neurons in the LDTg project to respiratory nuclei located in the brainstem, we examined the extent of LDTg cholinergic projections to brainstem respiratory nuclei using ChAT^cre^ Ai6 animals that received a unilateral injection of the cre-dependent adenovirus in the LDTg (AAV-hM3Dq-mCherry) (Figure 2J). The transfection rate was 83% (Figure 2K). We identified projections to the contralateral LDTg, RTN, postinspiratory complex (PiCo), preBötC and rostral ventral respiratory group (rVRG) (Figures 2L–2S), demonstrating that cholinergic neurons in the LDTg directly project to important nuclei responsible for modulating respiration.

### Characterization of distinct subpopulations of LDTg neurons in PD-mice

**Figure 3:**
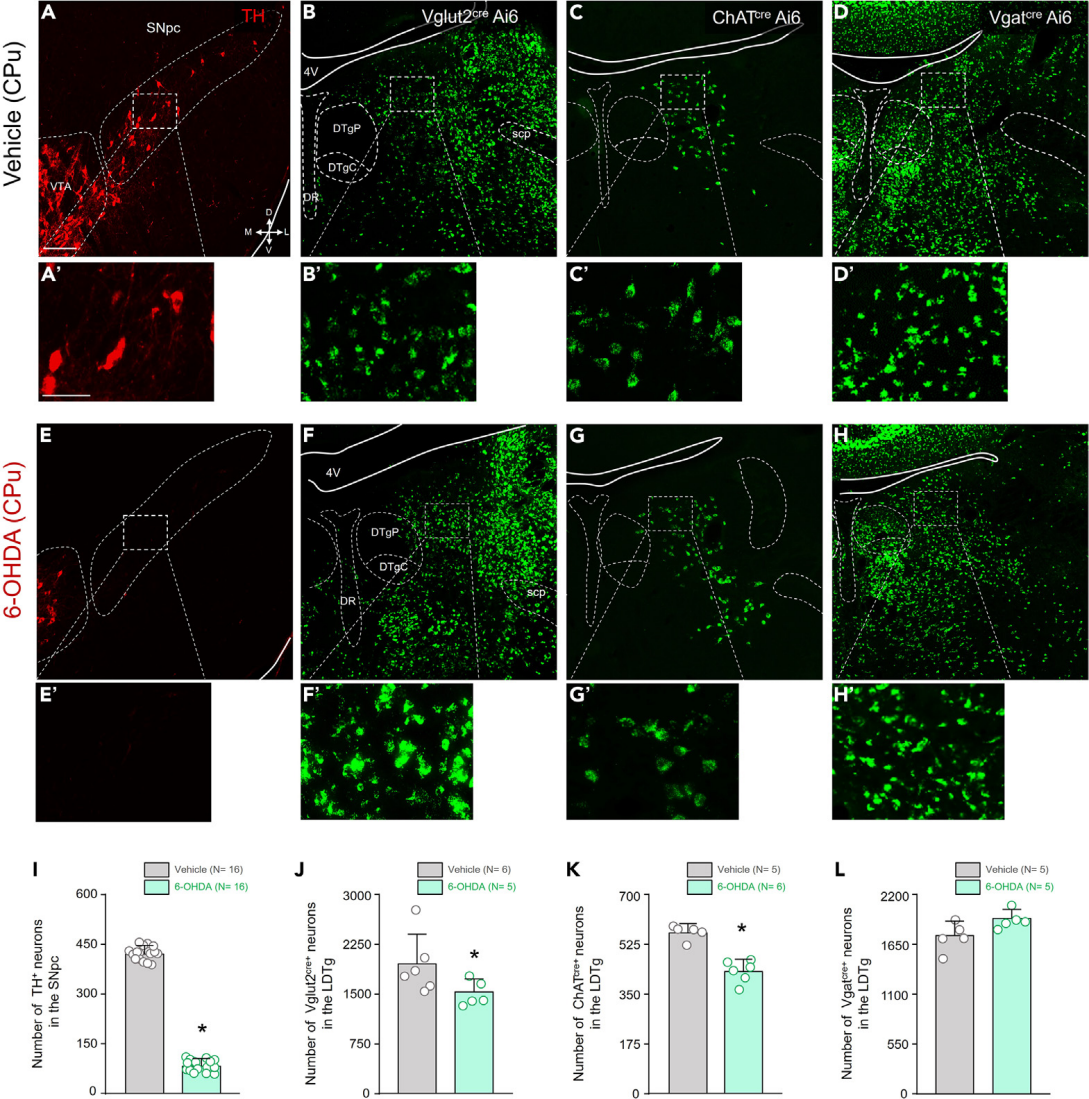
Quantification of the number of neurons in LDTg in Vglut2^cre^ Ai6, ChAT^cre^ Ai6 and Vgat^cre^ Ai6 mice that received bilateral injections of either vehicle or 6-OHDA into the CPu Data are represented as mean ± SD (A–D) Photomicrographs showing TH immunoreactivity in the SNpc (A), showing the fluorescence of Vglut2^cre^ Ai6 (B), ChAT^cre^ Ai6 (C), and Vgat^cre^ Ai6 (D) of mice that received bilateral CPu injection of vehicle in the CPu were analyzed 10 days after injection. (E–H) Photomicrographs showing TH immunoreactivity in the SNpc (E), showing the fluorescence of Vglut2^cre^ Ai6 (F), ChAT^cre^ Ai6 (G), and Vgat^cre^ Ai6 (H) of mice that received bilateral CPu injection of 6-OHDA into the CPu were analyzed 10 days after injection. (I–L) Quantification of the total number of neurons TH^+^ in the SNpc region (I), number of Vglut2^+^ neurons (J), number of ChAT^+^ neurons (K) and Vgat^+^ neurons (L) in LDTg in animals that received vehicle or 6-OHDA in CPu Abbreviations: 4V, 4th ventricle; DTgC, dorsal tegmental nucleus, central part; DTgP, dorsal tegmental nucleus, pericentral part; DR, dorsal raphe nucleus; scp, superior cerebellar peduncle. Scale bars: A = 500 μm (applies to A–H) and A’ = 100 μm (applies to A′– H′). Statistical analysis: ∗different from vehicle, *p* < 0.05, t test.

PD is marked by neurodegeneration specifically in the basal ganglia. As expected, in the PD-induced group, we observed a 79.6% reduction in dopaminergic neurons in the SNpc (PD: 86.2 ± 17.3 vs. Control: 423.8 ± 20.6 neurons, *p* < 0.001, t = 50.01, df = 30) (Figures 3A–3E and 3I). However, it is unknown whether the number of LDTg neurons is also impacted in PD mice. Therefore, in the subsequent experiments, we utilized the same type of animals as in the previous protocol to compare the number of distinct neuronal subtypes in the LDTg region between control and PD-animals. In LDTg, we noted a 23% decrease in cholinergic neurons (PD: 434.0 ± 38.9 vs. Control: 570.8 ± 26.1 neurons, *p* < 0.0001, t = 6.675, df = 9) (Figures 3C–3G and 3K), and 17% decrease in glutamatergic neurons (PD: 1531.0 ± 192.7 vs. Control: 1854.0 ± 193.2 neurons, *p* = 0.0219, t = 2.765, df = 9) (Figures 3B–3F and 3J) while we found no significant alteration in the number of GABAergic neurons (PD: 1940.0 ± 98.4 vs. Control: 1751.0 ± 158.7 neurons, *p* = 0.0538, t = 2.259, df = 8) (Figures 3D–3H and 3L).

**Figure 4:**
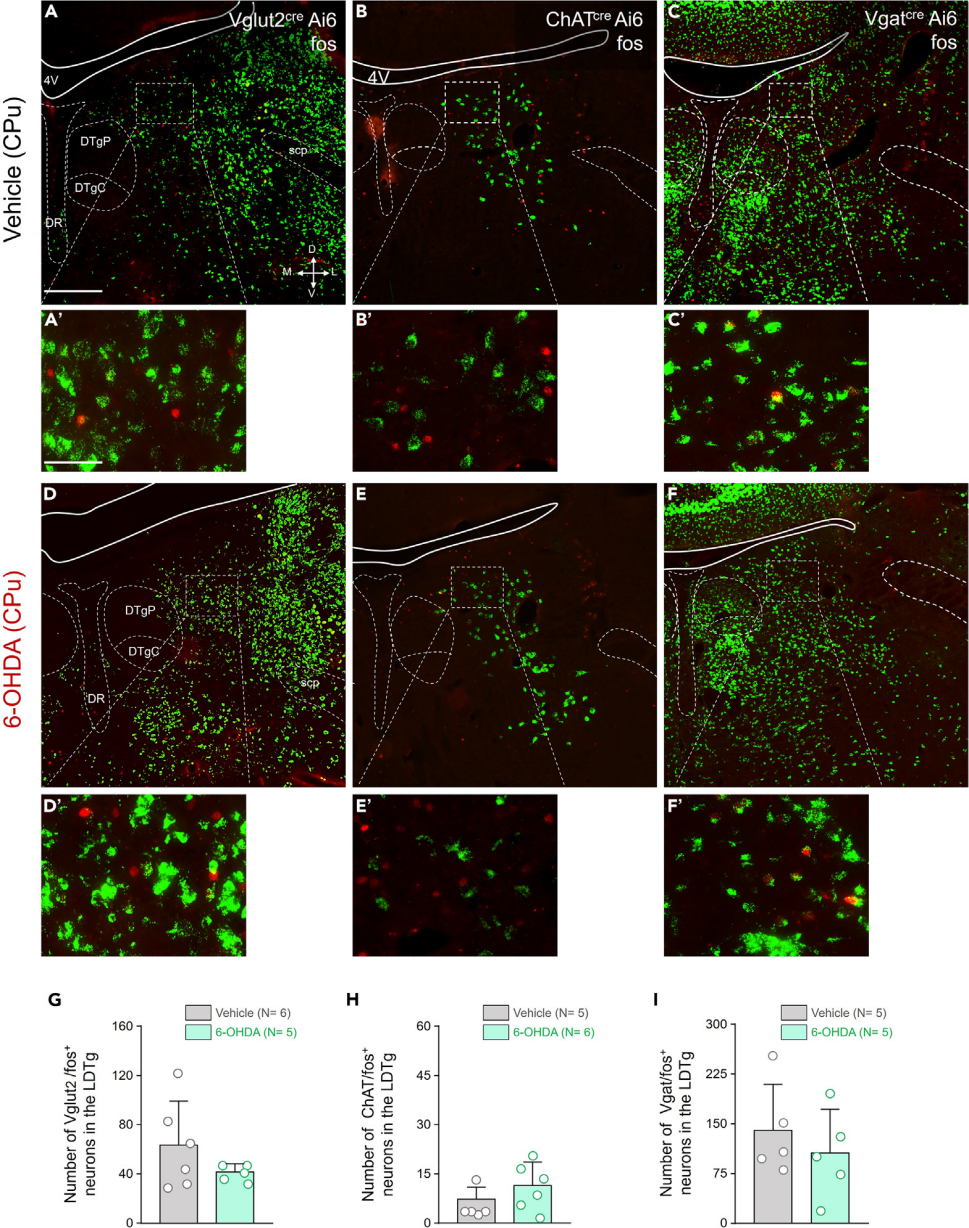
Expression of fos protein induced by hypercapnia stimulation in LDTg in Vglut2^cre^ Ai6, ChAT^cre^ Ai6, and Vgat^cre^ Ai6 mice that received bilateral injections of either vehicle or 6-OHDA into the CPu Data are represented as mean ± SD (A–F) Photomicrographs showing the fluorescence for Vglut2^cre^ Ai6, ChAT^cre^ Ai6 and Vgat^cre^ Ai6 and the immunofluorescence to protein fos, in mice that received vehicle (A, B, and C) or 6-OHDA in CPu (D, E, and F). (G–I) Number of G) Vglut2/fos^+^ neurons, H) ChAT/fos^+^ neurons and I) Vgat/fos^+^ neurons in LDTg in animals that received vehicle or 6-OHDA in the striatum. Abbreviations: 4V, 4th ventricle; DTgC, dorsal tegmental nucleus, central part; DTgP, dorsal tegmental nucleus, pericentral part; DR, dorsal raphe nucleus; scp, superior cerebellar peduncle. Scale bars: A = 500 μm (applies to A–F) and A’ = 100 μm (applies to A′–F′).

We next stimulated the central chemoreflex to characterize the activation of LDTg neurons and potential differences between groups that received either vehicle or 6-OHDA into the CPu. But we did not observe significant differences between the groups (Figure 4). To further validate our findings, we included an experimental control group exposed to room air for 180 min. In these animals, normoxia failed to induce fos expression in any cholinergic, GABAergic, or glutamatergic neurons in the LDTg (data not shown).

### Involvement of cholinergic neurons from the LDTg in the respiratory and sleep-wake cycle alterations in the different phases of the sleep-wake cycle in PD-mice

**Figure 5:**
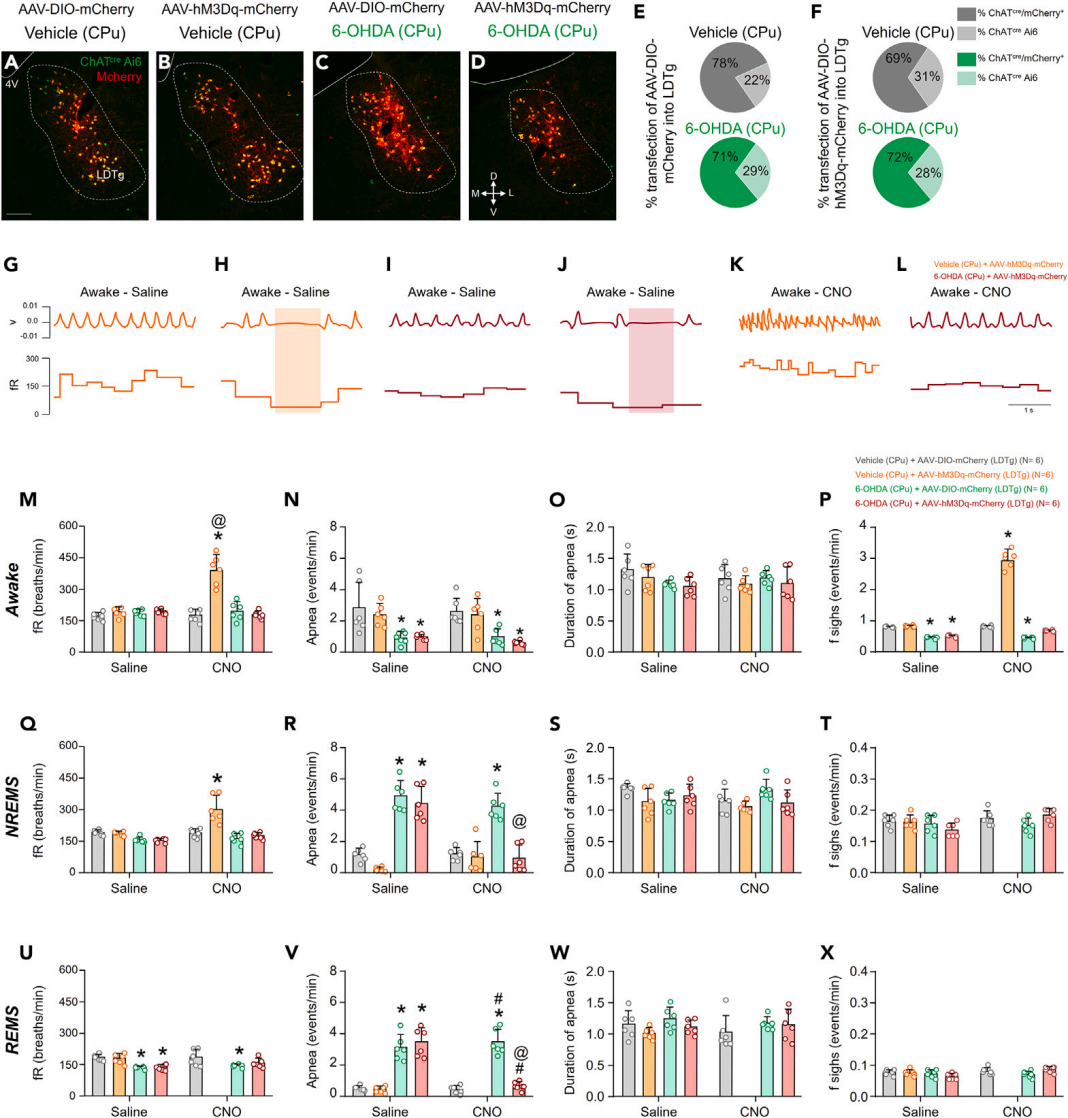
Effects of selective activation of LDTg-cholinergic neurons on respiratory dynamics across various phases of the sleep-wake cycle in mice subjected to bilateral injections of either vehicle or 6-OHDA into the CPu Data are represented as mean ± SD. (A–D) Representative images showing co-localization in the LDTg of fluorescence in cholinergic neurons (Ai6) and the associated adenovirus (MCherry) in animals receiving vehicle (A and B) or 6-OHDA (C and D) into the CPu. In images A and C, the adenovirus used was AAV-DIO-mCherry, and in B and D, it was AAV-hM3Dq-mCherry. (E and F) Percentage of infection of the AAV-DIO-mCherry (E) and the AAV-hM3Dq-mCherry (F) in mice receiving vehicle or 6-OHDA into the CPu. (G–J) In wake, examples of the recording of basal ventilation (G and I) and the apnea (H and J) in animals receiving vehicle (G and H) or 6-OHDA (I and J) into the CPu, the AAV-hM3Dq-mCherry in LDTg and saline ip. (K and L) In wake, examples of the recording of basal ventilation in animals receiving vehicle (K) or 6-OHDA (L) into the CPu, the AAV-hM3Dq-mCherry in LDTg and CNO ip. (M–P) Graphs showing the quantification of respiratory frequency (M), the quantification of the number of apneas (N), the quantification of apnea duration (O) and the quantification of the number of sigh (P) during wakefulness in animals receiving vehicle or 6-OHDA into CPu. (Q–T) Graphs showing the quantification of respiratory frequency (Q), the quantification of the number of apneas (R), the quantification of apnea duration (S) and the quantification of the number of sigh (T) during NREM sleep in animals receiving vehicle or 6-OHDA into CPu. (U–X) Graphs showing the quantification of respiratory frequency (U), the quantification of the number of apneas (V), the quantification of apnea duration (W) and the quantification of the number of sigh (X) during REM sleep in animals receiving vehicle or 6-OHDA into CPu. Abbreviations: 4V, ventricle; LDTg, laterodorsal tegmental nucleus; SNpc, substantia nigra pars compacta; VTA, ventral tegmental área; NREMS, NREM sleep; REMS, REM sleep; fR, respiratory frequency. Scale bars: A = 500 μm (applies to A–D). Statistical analysis: ANOVA ∗ different from Vehicle (CPu) + AAV-DIO-mcherry (LDT), #different from 6-OHDA (CPu) + AAV-DIO-mcherry (LDT) and @different from the same treatment with vehicle ip, p < 0.05.

To explore the specific role of cholinergic neurons in the LDTg we employed a chemogenetic approach and injected AAV-DIO-mCherry or AAV-hM3Dq-mCherry bilaterally into LDTg. We characterized four animal groups as follows: two groups of PD-induced mice injected with AAV-DIO-mCherry or AAV-hM3Dq-mCherry and two groups of control mice similarly injected with one or the other chemogenetic agent. Each experimental group was administered with either saline or CNO ip. at an interval of 1 day. First, we reconfirmed the reduction in the number of LDTg cholinergic neurons in the PD-induced group, which was consistent with the results described previously (PD: 433.3 ± 34.5 vs. Control: 604.2 ± 30.2 neurons, *p* < 0.0001, t = 9.122, df = 10) (data not shown). Then, we assessed the infection efficiency of the AAV injections in different groups which revealed a transduction rate exceeding 65% in all four groups (Figures 5A–5F). Furthermore, validation of our PD-induced model showed an approximate 80% of reduction in SNpc dopaminergic neurons (PD: 83.0 ± 14.8 vs. Control: 425.0 ± 28.3 neurons, *p* < 0.0001, t = 26.19, df = 10) (data not shown).

Plethysmography recordings of sleep-related breathing abnormalities in our 6-OHDA-PD model revealed a reduction in respiratory frequency during REM sleep (PD: 140.7 ± 6.6 vs. Control: 188.4 ± 15.0 breaths/minute, *p* = 0.0010, t = 4.164, df = 40) (Figure 5U). Interestingly, this reduction is attenuated when stimulating the cholinergic neurons in the LDTg through chemogenetics by CNO ip (PD mice, CNO: 162.3 ± 22.4 vs. Control mice, CNO: 188.3 ± 39.7 breaths/minute, *p* = 0.1724, t = 2.269, df = 40) (Figure 5U). Other analyses of this series of experiments showed no changes during NREM sleep in 6-OHDA-PD mice (PD mice, CNO:158.0 ± 13.1 vs. Control mice, CNO: 190.8 ± 12.9 breaths/minute, *p* = 0.2752, t = 2.061, df = 40) (Figure 5Q). We also did not observe any changes during wake in 6-OHDA-PD mice (PD mice, CNO:170.3 ± 17.8 vs. Control mice, CNO: 166.1 ± 28.3 breaths/minute, *p* > 0.9999, t = 1.165, df = 40) (Figure 5M). Interestingly, the chemogenetic activation of LDTg promoted a significant increase in respiratory frequency during NREM sleep in control mice (Control mice, CNO: 308.1 ± 66.4 vs. Control mice, vehicle: 189.4 ± 20.1 breaths/minute, *p* < 0.0001, t = 7.450, df = 40) and during wakefulness (Control mice, CNO: 390.1 ± 72.2 vs. Control mice, vehicle: 166.1 ± 28.3 breaths/minute, *p* < 0.0001, t = 11.25, df = 40) (Figures 5M and 5Q).

Additionally, we observed a decrease in the number of apneas during wakefulness in 6-OHDA-PD mice as previously described, which persisted during stimulation of cholinergic neurons in the LDTg (PD mice: 0.5 ± 0.1 vs. Control mice: 1.8 ± 0.2 apneas/minute, *p* = 0.0427, t = 2.837, df = 40) (Figure 5N). Surprisingly, when chemoactivating LDTg during NREMS, we observed a decreased number of apneas in 6-OHDA-PD mice, with no change in their duration (PD mice, CNO: 0.9 ± 0.8 vs. Control mice, CNO: 1.2 ± 0.4 apneas/minute, *p* > 0.9999, t = 0.5979, df = 40) (Figures 5R and 5S). Similarly, changes were observed during REMS (PD mice, CNO: 0.7 ± 0.2 vs. Control mice, CNO: 0.4 ± 0.2 apneas/minute, *p* > 0.9999, t = 0.7961 df = 40) (Figures 5V and 5W).

Furthermore, we observed a decrease in the number of sighs during in 6-OHDA-PD mice, as previously described, which persisted during the chemogenetic stimulation of cholinergic neurons in the LDTg (PD: 0.5 ± 0.04 vs. Control: 0.7 ± 0.04 sigh/minute, *p* = 0.0650, t = 2.637, df = 40) (Figure 5P). However, during NREMS and REMS, we also did not observe any changes in 6-OHDA-PD mice (PD: 0.1 ± 0.02 vs. Control: 0.1 ± 0.01 sigh/minute, *p* = 0.5004, t = 1.776, df = 40 in NREMS, and PD: 0.05 ± 0.06 vs. Control: 0.1 ± 0.06 sigh/minute, *p* = 0.5004, t = 1.776, df = 40 in REMS) (Figures 5T and 5X).

**Figure 6:**
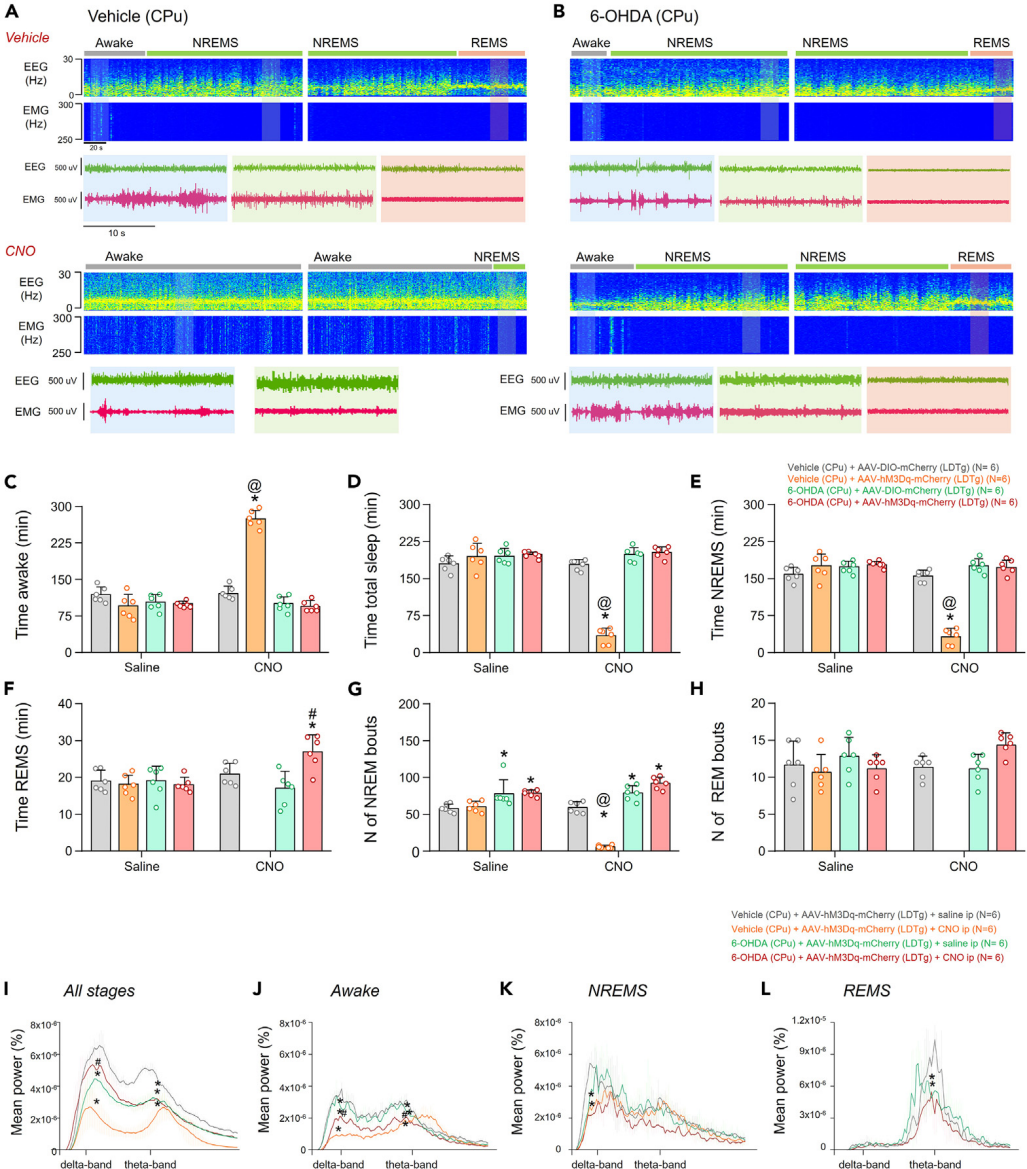
Involvement of LDTg cholinergic neurons in the sleep-wake cycle in mice receiving bilateral injections of either vehicle or 6-OHDA into the CPu Data are represented as mean ± SD. (A and B) Representative recordings of electroencephalographic and electromyographic waves in mice receiving vehicle (A) or 6-OHDA into the CPu (B), with vehicle or CNO ip. (C–F) Comparative data related to total wake time (C), total sleep time (D), NREM sleep time (E), and REM sleep time (F). (G and H) Comparative data related to the number of NREM (G) and REM (H) sleep episodes. (I–L) Spectrograms representing the different brain wave frequencies in the distinct phases of the sleep-wake cycle during saline or CNO ip recording for an hour (in all phases) (I), or only during wakefulness (J), NREMS (K), or REMS (L). Abbreviations: NREMS, NREM sleep; REMS, REM sleep; EEG, electroencephalogram; EMG, electromyogram. Statistical analysis: two-way ANOVA, Bonferroni post-test. ∗different from Vehicle (CPu) + AAV-DIO-mcherry (LDT), #different from 6-OHDA (CPu) + AAV-DIO-mcherry (LDT) and @different from the same treatment with vehicle ip, p < 0.05.

During bilateral selective DREADD chemogenetic stimulation of the LDTg in control animals (i.e., vehicle in the CPu) (Figure 6A), we observed more than 2-fold increase in wakefulness time compared to the same group without cholinergic neuron stimulation in the LDTg (Control mice, CNO: 276.2 ± 17.1 vs. Saline: 125.1 ± 13.1 min, *p* < 0.0001, t = 17.24, df = 40) (Figure 6C), resulting in a concurrent decrease in total sleep time (Control mice, CNO: 32.1 ± 16.2 vs. Saline: 176.7 ± 10.2 min, *p* < 0.0001, t = 23.15, df = 40) and NREMS time (Control mice, CNO: 32.1 ± 16.2 vs. Saline: 156.5 ± 11.1 min, *p* < 0.0001, t = 14.68, df = 40) (Figures 6D and 6E). Interestingly, these same animals did not exhibit REMS events (Figure 6F). In contrast, in 6-OHDA-PD mice showed an increased REMS time (PD mice, CNO: 27.1 ± 4.5 vs. Saline: 21.0 ± 2.9 min, *p* = 0.0153, t = 3.219, df = 40 (Figure 6F), an increase in the number of NREM bouts (PD mice, CNO: 92.3 ± 8.2 vs. Saline: 59.5 ± 7.6, *p* < 0.0001, t = 3.815, df = 40) and no differences in number of REM bouts (PD mice, CNO: 14.1 ± 1.4 vs. Saline: 11.3 ± 1.5, *p* = 0.1358, t = 2.371, df = 40) (Figures 6G and 6H).

As a control for our DREADD stimulation, we administered CNO injections to a group of control animals, with vehicle in CPu (*n* = 4) and 6-OHDA in CPu (*n* = 4) without viral injection. The results showed no significant changes in the duration NREMS in control (Control mice, saline: 155.5 ± 11.5 vs. CNO: 154.7 ± 12.1 min, *p* > 0.9999, t = 0.1029, df = 12) or in 6-OHDA-PD mice (PD mice: 182.2 ± 9.6 vs. CNO: 178.4 ± 6.5 min, *p* > 0.9999, t = 0.5297, df = 12), REMS in control (Control mice, saline: 19.7 ± 1.0 vs. CNO: 20.5 ± 2.0 min, *p* > 0.9999, t = 0.6055, df = 12) or in 6-OHDA-PD mice (PD mice: 21.9 ± 2.0 vs. CNO: 22.2 ± 1.5 min, *p* > 0.9999, t = 0.2381, df = 12) and in wakefulness in control (Control mice, saline: 125.7 ± 10.7 vs. CNO: 123.4 ± 10.8 min, *p* > 0.9999, t = 0.3233, df = 12) or in 6-OHDA-PD mice (PD mice: 95.8 ± 10.2 vs. CNO: 99.3 ± 7.0 min, *p* > 0.9999, t = 0.5064, df = 12).

### Involvement of LDTg cholinergic neurons in brain activity during sleep and wake in PD-animals

Power spectral analysis of electroencephalographic (EEG) activity during all vigilance states combined showed a noticeable difference between the groups of control animals that received CNO versus saline. There was a decrease in the amplitude of delta (Control mice, CNO: 2.85 × 10^−6^ ± 1.33 × 10^−6^ vs. saline: 5.82 × 10^−6^ ± 0.55 × 10^−6^%, *p* < 0.0001, t = 10.91, df = 2320) and theta power (Control mice, CNO: 2.73 × 10^−6^ ± 0.80 × 10^−6^ vs. saline: 4.19 × 10^−6^ ± 0.19 × 10^−6^%, *p* < 0.0001, t = 6.13, df = 2320) (Figure 6I). The same pattern of change in EEG activity was observed in the 6-OHDA-PD mice, in both delta frequency band (PD mice, CNO: 4.01 × 10^−6^ ± 0.75 × 10^−6^ vs. saline: 5.82 × 10^−6^ ± 0.55 × 10^−6^%, *p* < 0.0001, t = 6.63, df = 2320) and theta frequency band (PD mice, CNO: 3.16 × 10^−6^ ± 0.43 × 10^−6^ vs. saline: 4.19 × 10^−6^ ± 0.19 × 10^−6^%, *p* = 0.0002, t = 4.164, df = 2320) (Figure 6I). However, in 6-OHDA-PD mice treated with CNO, there was only a decrease in the theta power peak amplitude (PD-CNO: 3.10 × 10^−6^ ± 0.29 × 10^−6^vs. saline: 5.82 × 10^−6^ ± 0.55 × 10^−6^%, *p* = 0.0003, t = 4.029, df = 2320), showing that in these animals, there was an improvement in the amplitude of the delta wave when compared to 6-OHDA-PD mice and saline (PD mice, CNO: 5.31 × 10^−6^ ± 0.60 × 10^−6^ vs. saline: 4.02 × 10^−6^ ± 0.75 × 10^−6^%, *p* < 0.0001, t = 4.75, df = 2320, represented in the graph by #) (Figure 6I).

Power spectral analysis of EEG activity during wakefulness revealed a reduction in the amplitude of peak delta power in the control group after chemoactivation by CNO ip (Control mice, CNO: 0.98 × 10^−6^ ± 1.577e-007 vs. control AAV: 3.622 × 10^−6^ ± 0.27 × 10^−6^%, *p* < 0.0001, t = 16.76, df = 5) as well as a decrease in theta power (Control mice, CNO: 1.93 × 10^−6^ ± 0.28 × 10^−6^ vs. control AAV: 2.7 × 10^−6^ ± 0.13 × 10^−6^%, *p* = 0.0025, t = 8.305, df = 5) (Figure 6J). Similarly, during wakefulness, 6-OHDA-PD mice, presented an attenuated delta amplitude during chemoactivation of LDTg (PD mice, saline: 2.89 × 10^−6^ ± 0.52 × 10^−6^ vs. control mice + saline: 3.62 × 10^−6^ ± 0.27 × 10^−6^%, *p* = 0.0421, t = 4.401, df = 5) and theta waves (PD mice saline:2.23 × 10^−6^ ± 0.18 × 10^−6^ vs. control AAV: 2.77 × 10^−6^ ± 0.13 × 10^−6^%, *p* = 0.0036, t = 7.659, df = 5) (Figure 6J).

During the NREMS phase, differences were observed in animals that received CNO. In this case, there was a decrease in the amplitude of delta waves in animals that received vehicle into CPu (PD mice, CNO 2.85 × 10^−6^ ± 0.51 × 10^−6^ vs. saline: 5.28 × 10^−6^ ± 0.47 × 10^−6^%, *p* = 0.0011, t = 9.909, df = 5) (Figure 6K) and 6-OHDA in the CPu (PD mice, CNO 2.40 × 10^−6^ ± 0.33 10^−6^ vs. control mice, saline: 5.28 × 10^−6^ ± 0.47 × 10^−6^%, *p* = 0.0009, t = 10.21, df = 5) (Figure 6K).

Finally, regarding the REMS phase, the LDTg chemoactivation impacted the existence of this particular phase in control animals (Figure 6L). However, we observed an attenuated theta wave in 6-OHDA-PD mice (PD mice, saline: 4.55 × 10^−6^ ± 2.3 × 10^−6^%, *p* = 0.0194, t = 4.489, df = 5), which remained attenuated after LDTg chemoactivation (PD mice, CNO: 5.55 × 10^−6^ ± 1.94 × 10^−6^ vs. control mice, saline: 9.33 × 10^−6^ ± 0.97 × 10^−6^%, *p* = 0.0194, t = 4.489, df = 5) (Figure 6L).

## Discussion

In this study, we characterized the interplay between disturbances in sleep and respiratory functions in the 6-OHDA mouse model of PD. PD-animals spent less time awake and more time in NREMS, which was potentially explained by the increase in NREMS episodes. Additionally, these mice exhibited a reduced respiratory frequency and an increased number of apneas during NREMS and REMS. These changes could be attributed to neurodegeneration in a crucial region for awake-sleep cycle: the LDTg. The neurodegeneration specifically affected a subset of cholinergic neurons in this area. To restore sleep and/or respiratory function, we chemogenetically activated LDTg neurons in both control and PD-animals, resulting in a partial restoration of respiratory frequency and a decrease in the number of apneas during NREMS and REMS. Surprisingly, control animals that received LDTg stimulation spent significantly more time awake than all other groups, and this stimulation was sufficient to prevent REMS. Furthermore, the same control mice exhibited a significant increase in respiratory frequency, indicating that the LDTg might indeed play a crucial role in the interplay between respiration and sleep.

### Parkinson disease, sleep, and breathing

In the experimental model used in the present study, we observed respiratory changes, manifested as a reduction in fR, as reported on several occasions before.^21^^,^^34^^,^^35^ However, it remained unknown whether these changes correlated with different phases of the sleep-wake cycle. In this study, we observed that in the PD model, there is a decrease in fR and an increase of apneas during both NREMS and REMS. These animals exhibited reduced wakefulness, spent more time in NREMS which was reflected in an increased number of NREMS episodes.

One of the neurotransmitters crucial for wakefulness is dopamine, a neurotransmitter affected in PD. In this study, we used animals with approximately 80% damage to dopaminergic neurons in the SNpc. Importantly, animals that received 6-OHDA into the CPu also showed a 25% degeneration of cholinergic neurons in the LDTg. The cholinergic areas known as LDTg and PPTg both play a role in promoting wakefulness and cognitive functions. They are known to be responsible for inhibiting slow cortical oscillations during NREMS and are activated during REMS.^28^^,^^36^ Here, we show that cholinergic neurons of LDTg send direct projections to areas involved in breathing regulation such as RTN, PiCo, preBötC, and rVRG. The reduction in the number of these neurons suggests a potential link between this degeneration and alterations in breathing and sleep in this experimental model.

Optogenetic activation of the cholinergic neurons in the LDTg during NREMS is known to increase the probability of transitioning to REMS.^37^ To reproduce and extend these findings, we conducted chemogenetic stimulation of these neurons. Surprisingly, in contrast to the results reported by Van Dort and colleagues, we observed a reduction in REMS in control animals with chemogenetic stimulation. These animals spent approximately three times more in awake state compared to the non-stimulated group. Conversely, in PD animal model, we found that stimulation could increase the number of NREM bouts. Moreover, the respiratory rate and number of apneas were restored to control levels in our PD model.

To evaluate sleep quality and depth, we conducted a spectral power analysis, which allows for detailed exploration of sleep microstructure.^38^^,^^39^^,^^40^^,^^41^^,^^42^ Our findings revealed that LDTg stimulation during wakefulness in animals that received vehicle into the CPu led to a significant reduction in theta wave power by up to 70% and delta wave power by 27%. In animals that received 6-OHDA into the CPu, there was 80% reduction in both delta and theta waves, suggesting a state of stress, anxiety, and cognitive impairment.^42^^,^^43^^,^^44^^,^^45^ During NREMS, significant differences were observed primarily in the group that received both 6-OHDA into the CPu and chemoactivation of LDTg by CNO ip injection, showing a 45% reduction in delta waves. This reduction may indicate compromised sleep quality or non-restorative sleep, often associated with insomnia, and potential cognitive decline.^46^^,^^47^^,^^48^ Additionally, during REMS, animals that received 6-OHDA into the CPu showed a decrease in theta wave power, which persisted with LDTg chemoactivation. This finding could indicate sleep fragmentation, deteriorated REMS quality, and memory disturbances,^49^^,^^50^ which are also relevant to PD.^51^

### The LDTg and PD

The LDTg is a cluster of neurons located in the medio-lateral portion of the pons, ventrally to the transition of aqueduct/fourth ventricle.^52^ Traditionally identified as a cholinergic region, the phenotype of this area is complex, with various overlapping neuron populations. Importantly, it contains a dense population of both glutamatergic and GABAergic neurons.^27^^,^^52^^,^^53^ Here, we showed that the quantity of glutamatergic and GABAergic neurons in the LDTg region is approximately equal (∼1961 and ∼1698, respectively). This contrasts with the findings of Luquin and colleagues, who observed in rats that the GABAergic neuronal population was larger than the glutamatergic population, suggesting a neuronal distinction among rodent species.^54^ Additionally, our findings revealed that around 22% of glutamatergic neurons in the LDTg were also cholinergic, making it the smallest of the three analyzed neuronal populations. In the same study, Luquin et al. noted that 5 to 10% of glutamatergic neurons in the LDTg were also cholinergic.^54^ Steinkellner and colleagues, focusing on mice, observed that the cholinergic mesopontine neurons mostly expressed reactivity to the vesicular glutamate transporter, classifying these neurons as glutamatergic. However, they also found rare cholinergic neurons that were GABAergic, aligning with the results of the present study.^55^

In PD, motor symptoms such as postural and gait changes, are frequently linked to alterations in the cholinergic systems. Patients exhibit a substantial reduction, approximately 30%, in acetylcholinesterase activity in the brain, a factor strongly correlated with dementia, impaired cognitive function, gait disturbances, an increased propensity for falls, and sleep disorders. Additionally, there is evident degeneration in the cholinergic system in the brain.^30^^,^^56^^,^^57^^,^^58^^,^^59^^,^^60^ Our recent PD-model study demonstrated a reduction in cholinergic neurons in the brainstem.^34^^,^^61^ Our data showed a 23% degeneration of cholinergic and 17% glutamatergic neurons in the LDTg in animals that received 6-OHDA into the CPu, with no significant changes in the GABAergic neurons. Another important cholinergic area in the pons is the pedunculopontine tegmental nucleus (PPTg), which, like the LDTg, plays a role in sleep regulation but remains unaffected in our PD model.^62^

In conclusion, this study reveals neurodegeneration in specific brain regions in the mouse model of PD induced by 6-OHDA, impacting normal fR and sleep patterns. Selective stimulation of the remaining cholinergic LDTg neurons results in the normalization of state-dependent respiratory parameters indicating a fundamental role in modulating respiration and sleep in this experimental model. Furthermore, it alters entire sleep architecture in control animals, underscoring the essential role of the LDTg in sleep quality and regulating REM sleep.

### Limitations of the study

Our study presents some limitations that should be highlighted. Although the 6-OHDA-induced PD experimental model reproduces several motor and non-motor symptoms observed in patients, it does not fully reflect the pathological accumulation of alpha-synuclein or the formation of Lewy bodies, which are key pathological features of human PD. Furthermore, the sleep analysis was conducted manually, potentially introducing variability in the classification of sleep stages. Another limitation is that our analysis was restricted to the light phase of the sleep cycle, which may have missed capturing relevant changes occurring in other phases of the sleep-wake cycle. Future studies utilizing automated methods and more comprehensive analyses across different sleep phases may provide a deeper understanding of the observed changes.

## Resource availability

### Lead contact

Further information and requests for resources and reagents should be directed to and will be fulfilled by the lead contact, Prof. Ana C. Takakura (takakura@icb.usp.br).

### Materials availability

This study did not generate any reagents. The breeding animals were acquired from Jackson Laboratory and were generated at Department of Physiology and Biophysics at the University of Sao Paulo or at Seattle Children’s Research Institute Animal Facility. The adenovirus used is from Addgene.

### Data and code availability

Data: The data generated or analyzed during this study are not publicly available in repositories. They can be obtained upon request from the lead contact Ana C. Takakura via takakura@icb.usp.br.
Code: No specific code was used in the development of this study. Any additional details about the analysis process can be provided by the lead contact upon request.
Other items: Experimental protocols and supplementary materials are available upon request from the lead contact.

## Acknowledgments

Our studies are supported by the following grants: 10.13039/501100001807São Paulo Research Foundation (FAPESP; grants: 2021/09768-5 to A.C.T.; 2015/23376-1 to T.S.M.; fellowships: 2021/05647-9 to N.C.M.; 2022/07705-9 to A.C.T.). This study was financed in part by the Coordenação de Aperfeiçoamento de Pessoal de Nível Superior - Brasil (CAPES) - Finance Code 001 and Conselho Nacional de Pesquisa (CNPq fellowships: 200715/2022-4 to N.C.M.; 306418/2023-1 to T.S.M.; 306580/2023-3 to A.C.T.).

## Author contributions

T.S.M., J.-M.R., and A.C.T. designed the experiments; N.C.M. collected the data; N.C.M., L.M.O., F.K., and A.C.T. analyzed the data; N.C.M., L.M.O., T.S.M., and A.C.T. wrote the paper. All authors approved the final version of the manuscript.

## Declaration of interests

The authors declare no competing interests. The illustrations and graphical abstract were partially created with BioRender.com.

## STAR★Methods

### Key resources table

**Table:** 

REAGENT or RESOURCE	SOURCE	IDENTIFIER
**Antibodies**		

mouse anti-TH	Millipore	Cat# MAB 318; RRID: AB_2201528
rabbit anti-fos	Cell Signaling	Cat# 2250; RRID: AB_2247211
goat anti-ChAT	Millipore	Cat# AB 144P; RRID:AB_2079751
donkey anti-mouse Alexa 594	Jackson Immuno Research Laboratories	Cat# 715-585-151; RRID: AB_2340855
donkey anti-rabbit Alexa 594	Jackson Immuno Research Laboratories	Cat# 711-585-152; RRID: AB_2340621
donkey anti-rabbit Alexa 647	Jackson Immuno Research Laboratories	Cat# 711-605-152; RRID: AB_2492288
donkey anti-goat Alexa 594	Jackson Immuno Research Laboratories	Cat# 705-586-147; RRID: AB_2340434
donkey anti-goat Alexa 647	Jackson Immuno Research Laboratories	Cat# 705-605-003; RRID: AB_2340436

**Bacterial and virus strains**		

pAAV-hSyn-DIO-hM3D(Gq)-mCherry	ADDGENE	Cat# AAV1; 44361-AAV1; RRID: Addgene_44361
pAAV-hSyn-DIO-mCherry	ADDGENE	Cat# AAV1; 50459-AAV1; RRID: Addgene_50459

**Chemicals, peptides, and recombinant proteins**		

Clozapine-N-oxide	MedChemExpress	CAT#HY-17366
6-hydroxydopamine hydrochloride (6-OHDA)	Sigma	CAT#28094-15-7
L-ascorbic acid	Synter	CAT#A1022.01.AG
Isoflurane	Cristália	CAT#22110481

**Experimental models: Organisms/strains**		

Mouse: B6; 129S6-Chattm2(cre)Lowl/J (ChAT-IRES-Cre)	Jackson Laboratory	IMSR_JAX:006410
Mouse: Slc17a6tm2(cre)Lowl/J (Vglut2-ires-cre)	Jackson Laboratory	IMSR_JAX:016963
Mouse: Slc32a1tm2(cre)Lowl/J (Vgat-ires-cre)	Jackson Laboratory	IMSR_JAX:016962
Mouse: B6.Cg-Gt(ROSA)26Sortm6(CAG-ZsGreen1)Hze/J (Ai6)	Jackson Laboratory	IMSR_JAX:007906

**Software and algorithms**		

GraphPad Prism	GraphPad Software	RRID:SCR_002798; https://www.graphpad.com/
Iox 2.10.5.35	EMKA Technologies	https://www.emkatech.com/product/iox2-software/
LabChat 8	ADInstruments	https://www.adinstruments.com/support/software
ImageJ	ImageJ	https://imagej.nih.gov/ij/

### Experimental model and study participant details

#### Ethical statement

We declare that animals were handled in accordance with ethical requirements enacted by the Universidade de São Paulo and Seattle Children’s Research Center.

#### Animals

All experiments were performed in 60 adults C57/Bl6 mice of either sex (18-25g).

We used in our experiments animals from breeding: Vgat-IRES-cre^(+/+)^ (028863), Vglut2-IRES-cre^(+/+)^ (016962) or ChAT-IRES-cre^(+/+)^ (0206410) dam mice crossed with homozygous mice for the expression of cre-dependent fluorescent green protein (ZsGreen1; Ai6) (007906) inserted in the ROSA26 locus. The experiments were performed in Vgat^cre^ Ai6, Vglut2^cre^ Ai6 and ChAT^cre^ Ai6 produced from the pair of breeders.

The animals were housed in a temperature-controlled chamber at 24°C and light/dark cycle-controlled (12:12 h). In addition, all animals had free access to water and food. All experimental and surgical procedures were conducted in accordance with the National Institutes of Health and to defined guidelines by the Institutional Animal Care and Use Committee at the University of São Paulo (USP) (protocol number: 6641200919 and 8760150318) and Seattle Children’s Research Institute (SCRI) (protocol number: IACUC00058). The breeding animals were acquired from Jackson Laboratory and were generated at Department of Physiology and Biophysics at the University of Sao Paulo or at Seattle Children’s Research Institute Animal Facility and were randomly assigned to experimental groups.

### Method details

#### 6-OHDA injections

For selective chemical lesions of dopaminergic neurons in the SNpc, the animals were fixed in a stereotaxic apparatus (Kopf model 1760), one injection was made in each cerebral hemisphere of 6-OHDA (6-hydroxydopamine hydrochloride; Sigma, Saint Louis, MO, USA; 10 μg/μL; 0.5 μL; bilaterally into the striatum - CPu) or vehicle (1μg of ascorbic acid in 1μL of 0.9% saline, 0.5 μL; bilaterally into the CPu) while the mice were under general anesthesia induced by 2.0–2.5% inhaled isoflurane balanced in 100% oxygen. We used the following coordinates to reach the striatum region: 0.0 mm rostral to the bregma, 2.1 mm lateral to the midline and 3.0 mm ventral to the dura-mater. The injections were performed through a single-barrel metal needle with an external tip coupled to a Hamilton’s syringe.

#### Adenovirus injections

ChAT^cre^ Ai6 animals were anesthetized with isoflurane (2.0–2.5% balanced in 100% oxygen) and fixed in a stereotaxic apparatus (Kopf model 1760). After cranial opening, unilateral or bilateral injection of the AAV-hSyn-DIO-hM3Dq-mCherry (DREADD, 44361-AAV1) or AAV-hSyn-DIO-mCherry, 30 nL each, were performed using a glass micropipette with an internal tip diameter of 20 μm attached to the Picospritzer equipment. We used the following coordinates to reach the LDTg region: 0.5 mm caudal to the lambda, 0.4 mm lateral to the midline and 3.1 mm ventral to the dura-mater. Twenty days after the AAV-mCherry injection, the animals were deeply anesthetized and perfused for immunohistochemical analysis. We administered clozapine (1 mg/kg diluted in saline solution, intraperitoneally - ip), leading to the formation of the metabolite CNO, which selectively activates receptors expressed in cells infected by the AAV-hM3Dq-mCherry virus.

#### Respiratory frequency

Functional experiments were obtained through whole-body plethysmography (EMKA Technologies or Buxco, Sharon, Connecticut, CT, USA). This method involves a sealed and constantly ventilated chamber, where the animal is placed, and temperature and humidity are kept stable (±0.5°C and ±10%, respectively). Different pressure transducers record small changes in pressure inside the chamber. System calibration was performed by injecting a known volume of air (1 mL) into each chamber. The plethysmography chamber was continuously ventilated with 1–2 L/min of air (21% O_2_ balanced with N_2_) and the respiratory frequency (f_R_, breaths/min) was measured. The flow controllers were adjusted to 21% O_2_ balanced with N_2_ in normoxia. In the recording, an apnea event was defined as the cessation of respiratory activity lasting longer than two ventilation cycles beyond a normal eupneic breath. A sigh was identified when the inspiratory peak increased twice the amplitude of a eupneic breath.

#### Hypercapnia

Different groups of animals that express the green fluorescent protein in cholinergic, glutamatergic or GABAergic cells (i.e., ChAT^cre^ Ai6, Vglut2^cre^ Ai6 and Vgat^cre^ Ai6) and received vehicle or 6-OHDA injections bilaterally into the striatum were placed in a plethysmography chamber for at least 2 h to acclimate and exposed for 3 h to hypercapnia (7% CO_2_, 21% O_2_, balanced with N_2_) challenge. As control, 4 animals of each group, were exposed to room air under the same conditions. Following hypercapnia or normoxia/normocapnia, the animals were anesthetized with sodium thiopental (60 mg/kg, ip) and immediately perfusion fixed.

#### EEG/EMG recordings

Adult ChAT^cre^ Ai6 mice were used for this set of experiments and all surgical procedures were performed under aseptic conditions. Following anesthesia with isoflurane (2.0–2.5% in 100% O_2_), the mice were positioned in a stereotaxic frame (David Kopf Instruments, Tujunga, CA) to ensure head immobilization and stability. Subsequently, a local anesthetic (bupivacaine, 6 mg/kg) was administered subcutaneously. The animals then underwent a craniotomy, creating a 0.5 mm diameter perforation on both sides near the midline of the parietal bone. In this region, the following implants were carefully positioned: two silver electrodes for electroencephalogram (EEG) recording, visually identified in the parietal bone region between the hippocampus and cortex (one on each side of the skull), secured with cyanoacrylate glue and dental cement; two silver wire electrodes for electromyogram (EMG) recording in the neck muscle on the right side; one reference silver electrode on the occipital bone, used to minimize EEG wave noise; and one ground electrode, placed subcutaneously in the anterior chest region.

After the electrode implantation, the skin was sutured, and the animals were placed in separate cages to recover overnight. Following all surgical procedures, the animals received a dose of ketoprofen (7 mg/kg, subcutaneous) for two consecutive days.

On the day of the experiment, the animals were first connected to the data acquisition equipment and acclimatized in the plethysmography chamber for 1 h. EEG and EMG recordings were conducted continuously within the plethysmography chamber for 6 h, starting at 9:00 a.m. Concurrent video, EEG, EMG and respiration were recorded with a PowerLab 8/35 data acquisition system using LabChart 8 software (ADInstruments). A 100X preamplifier was connected directly to the electrode headmount. All electrophysiology signals were acquired at a sampling rate of 1KHz. The EEG traces were processed postop with a 1–70 Hz bandpass filter, the EMG with a 10 Hz high-pass filter, and the respiration signal with a 3 Hz high-pass filter. Data analysis was conducted using LabChart8 software. EEG/EMG recordings from the neck, along with video recordings were analyzed offline to score different states of vigilance of the animals during recording.

Among quantitative EEG methodologies, power spectral density (PSD) analysis is the predominant technique, providing an estimation of the power of the input signal against frequency and facilitating the investigation of sleep microarchitecture. This approach is used to evaluate the depth and quality of sleep, as well as levels of drowsiness.^38^^,^^39^^,^^40^ For our analysis, power spectral analyses were conducted on recordings segmented into 5-s epochs using Fast Fourier Transform (FFT). The quantitative analysis of the sleep-wake cycle was performed using intracranial local field potential (LFPs) from the EEG electrodes, muscle tone recordings from EMG electrodes, and behavioral activity scored from video recording. Wakefulness was identified by the presence of active muscle tone in the EMG, accompanied by exploratory behaviors, automatic movements, or stationary standing, with EEG frequencies characterized by low amplitude and high frequency. NREM sleep was identified by a decrease in muscle tone, with the animal lying down, immobile, and with closed eyes, and EEG frequencies characterized by medium amplitude and low frequency. REM sleep was identified by a muscle atonia, a lying-down posture, complete immobility, and EEG frequencies characterized by low amplitude and high frequency.^41^^,^^63^^,^^64^^,^^65^^,^^66^ EEG waveform differences were analyzed using the long-term spectrograms from power density of the EEG channels, employing custom-designed functionalities within the LabChart8 software (Hann window, FFT 4k and overlap 50%) (Ng et al., 2022).Offline analysis of the sleep-wake cycle was performed in a blinded manner by a single experimenter.

#### Histology

At the end of the experiments, the animals were deeply anesthetized with sodium thiopental (60 mg/kg, ip) and perfused through the left cardiac ventricle with phosphate buffer saline (PBS - pH 7.4), followed by formaldehyde (4% at 0.1 M phosphate buffer, pH 7.4). The brains were removed and stored in this fixative for 4 h at 4°C and transferred to a dehydrating solution for 8 h (sucrose 20%). Brain slices (30 μm thick) were performed using a microtome (Leica SM2010R) and stored in an antifreeze solution (cryoprotectant: 20% glycerol, 30% ethylene glycol in 50 mM phosphate, pH 7.4) which preserves the brain tissue for morphological analysis and quantification.

By immunofluorescence technique, tyrosine hydroxylase (TH) was detected using a polyclonal mouse anti-TH antibody (MAB 318; Millipore; 1:1000); fos protein was detected using a polyclonal rabbit anti-fos antibody (MAB 9F6; Cell Signaling; 1:5000); and choline acetyltransferase (ChAT) was detected using a polyclonal goat anti-ChAT antibody (AB 144P; Millipore; 1:500) diluted in PBS containing normal donkey serum (017-000-121; 1%; Jackson Immuno Research Laboratories) and Triton X-0.3% and incubated for 24 h. The sections were subsequently washed in PBS and incubated for 2 h in the donkey anti-mouse Alexa 594 (715-585-151; Jackson Immuno Research Laboratories; 1–500) or 647 (715-605-151; Jackson Immuno Research Laboratories; 1–500) antibody for TH; donkey anti-rabbit Alexa 594 (711-585-152; Jackson Immuno Research Laboratories; 1–500) or 647 (711-605-152; Jackson Immuno Research Laboratories; 1–500) antibody for fos and donkey anti-goat Alexa 594 (705-586-147; Jackson Immuno Research Laboratories; 1–500) or 647 (705-605-003; Jackson Immuno Research Laboratories; 1–500) antibody for ChAT.

Brain sections have been blinded analyzed using a fluorescence microscope (ZeissAxioskop A1, Oberkochen, Germany) for i) counting cholinergic neurons with mCherry^+^ in LDTg, ii) evaluate the expression of fos protein, anterograde projections, and cholinergic, glutamatergic, and GABAergic neurons in LDTg region, and iii) evaluate the expression of fos protein in the RTN. The analysis was performed as follows: 1) LDTg - 5 sections located in the medial region of the pons and lateral to the mesencephalic aqueduct were analyzed (bregma: −4.95 to −5.31mm), the limit for defining LDTg were the fourth ventricle and the periaqueductal gray matter (PAG) in the superior boundary in more caudal or more rostral sections, respectively, 2) SNpc: 5 sections located in the ventral quadrant of the midbrain and lateral to the VTA (bregma: −3.28 to −3.64 mm), the limit for defining SNpc were reticular substantia nigra, medial leminiscu and VTA, 3) RTN: 3 sections rostral from the caudal end of the facial nucleus (bregma: −6.34 to −6.52 mm); 4) rostral division of ventral respiratory group (rVRG): 3 sections under the nucleus Ambiguus compact and lateral to inferior olive, caudal to the preBötC (bregma: −7.19 to −7.37 mm); 5) preBötC: 2 sections, in the ventrolateral quadrant of the medulla, the dorsal border is ventral to the semi-compact division of the nucleus Ambiguus and it is ventral border is parallel to the dorsal boundary of the inferior olive (bregma: −6.95 to −7.04 mm); 6) Postinspiratory Complex (PiCo): 2 sections, medial to the rostral portion of nucleus Ambiguus, the last section with the caudal end of the facial motor neurons and the rostral portion of the inferior olives is the section that contains the rostral portion of PiCo (bregma: −6.93 to −7.02 mm).

### Quantification and statistical analysis

The statistical analysis was performed using the GraphPad Prism (version 8, GraphPad Software). Experiments were randomized. Sample sizes were not predetermined using statistical methods. The data were plotted as mean ± SD. All datasets were tested for identify outliers using ROUT test, with Q = 1%. For statistical analysis, test-t student was used for comparisons between two groups. Two-way analysis of variance (ANOVA) followed by the Bonferroni’s test was used to compare multiple groups. The samples are equivalent to the number of animals used. All statistical details can be found in the figure legends. For each analysis, *p* < 0.05 was considered statistically significant.
